# Immunological features of patients affected by Barraquer-Simons syndrome

**DOI:** 10.1186/s13023-019-1292-1

**Published:** 2020-01-10

**Authors:** Fernando Corvillo, Giovanni Ceccarini, Pilar Nozal, Silvia Magno, Caterina Pelosini, Sofía Garrido, Alberto López-Lera, Manuela Moraru, Carlos Vilches, Silvia Fornaciari, Sabrina Gabbriellini, Ferruccio Santini, David Araújo-Vilar, Margarita López-Trascasa

**Affiliations:** 1 Complement Research Group, Hospital La Paz Institute for Health Research (IdiPAZ), La Paz University Hospital, Paseo de la Castellana, 261, 28046 Madrid, Spain; 2 Center for Biomedical Network Research on Rare Diseases (CIBERER U754), Madrid, Spain; 3 Obesity and Lipodystrophy Centre at the Endocrinology Unit, University Hospital of Pisa, Pisa, Italy; 4 Unit of Immunology, La Paz University Hospital, Madrid, Spain; 5 Immunogenetics and Histocompatibility, Instituto de Investigación Sanitaria Puerta de Hierro, Madrid, Spain; 6 Immunogenetics laboratory, University Hospital of Pisa, Pisa, Italy; 7 Thyroid and Metabolic Diseases Unit (U.E.T.eM.), Centro Singular de Investigación en Medicina Molecular e Enfermidades Crónicas (CIMUS-IDIS), School of Medicine, Universidad de Santiago de Compostela, Santiago de Compostela, Spain; 8 Universidad Autónoma de Madrid, Madrid, Spain

**Keywords:** Complement system, Lipodystrophy, Barraquer-Simons syndrome, C3 nephritic factor, Autoimmunity, Acquired partial lipodystrophy

## Abstract

**Background:**

C3 hypocomplementemia and the presence of C3 nephritic factor (C3NeF), an autoantibody causing complement system over-activation, are common features among most patients affected by Barraquer-Simons syndrome (BSS), an acquired form of partial lipodystrophy. Moreover, BSS is frequently associated with autoimmune diseases. However, the relationship between complement system dysregulation and BSS remains to be fully elucidated. The aim of this study was to provide a comprehensive immunological analysis of the complement system status, autoantibody signatures and HLA profile in BSS. Thirteen subjects with BSS were recruited for the study. The circulating levels of complement components, C3, C4, Factor B (FB) and Properdin (P), as well as an extended autoantibody profile including autoantibodies targeting complement components and regulators were assessed in serum. Additionally, HLA genotyping was carried out using DNA extracted from peripheral blood mononuclear cells.

**Results:**

C3, C4 and FB levels were significantly reduced in patients with BSS as compared with healthy subjects. C3NeF was the most frequently found autoantibody (69.2% of cases), followed by anti-C3 (38.5%), and anti-P and anti-FB (30.8% each). Clinical data showed high prevalence of autoimmune diseases (38.5%), the majority of patients (61.5%) being positive for at least one of the autoantibodies tested. The HLA allele DRB1*11 was present in 54% of BSS patients, and the majority of them (31%) were positive for *11:03 (vs 1.3% allelic frequency in the general population).

**Conclusions:**

Our results confirmed the association between BSS, autoimmunity and C3 hypocomplementemia. Moreover, the finding of autoantibodies targeting complement system proteins points to complement dysregulation as a central pathological event in the development of BSS.

## Background

Lipodystrophies are a heterogeneous group of rare diseases characterized by loss of adipose tissue. They can be divided into generalized, partial or localized depending on the extent of fat loss. Additionally, both, generalized and partial forms can be classified as inherited and acquired [1]. Barraquer-Simons syndrome (BSS) (ORPHA: 79087), is an acquired form of partial lipodystrophy, characterized by bilateral symmetrical loss of adipose tissue that begins in the face and may variably spreads to neck, shoulders, arms and trunk, keeping intact the adipose tissue of the lower extremities [2]. Sometimes, after puberty, mainly in women, adipose tissue is hypertrophic in the lower extremities, causing a regional disproportion. Females are more affected than males with a ratio of 4:1 [3, 4]. There is usually no family history of lipodystrophy. Onset of the disease usually occurs during childhood or adolescence, sometimes after viral infections [5]. As opposed to other types of lipodystrophies, metabolic diseases and associated comorbidities appear less common in patients with BSS [4, 5], though some of them can present severe metabolic complications [6]. Several reports have shown the association of BSS with autoimmune diseases in a minority of patients, in particular systemic lupus erythematosus and dermatomyositis. Other autoimmune diseases that are less frequently associated with BSS include autoimmune thyroiditis, localized scleroderma, idiopathic thrombocytopenic purpura and Sjögren’s syndrome, among others [4, 5, 7].

A common feature among patients with BSS is the C3 hypocomplementemia [5]. Sissons et al. [8] provided a comprehensive study of the association of BSS with complement dysregulation. In that study, most patients with C3 consumption had detectable levels of an IgG/IgM autoantibody called C3 nephritic factor (C3NeF). C3NeF stabilizes the enzymatic complex C3 convertase (C3bBb), which causes unopposed activation of the alternative pathway (AP) of the complement system [9]. The presence of this autoantibody has been associated with a rare entity named C3 glomerulopathy (C3G) [10]. C3G is a kidney disease characterized by C3 predominant staining and minimal or absent immunoglobulin staining observed on renal biopsy by immunofluorescence [11]. The incidence of C3G is approximately 1 per million per year [12]. C3G is divided into dense deposit disease (DDD) and C3 glomerulonephritis (C3GN), based on electron microscopy findings [13]. The most important adverse outcome associated with the diagnosis of C3G is progression to end-stage renal disease, which occurs within 10 years from diagnosis in ~ 70% of affected children and 30–50% of affected adults [13]. Concerning patients with BSS, 20% of the them eventually develops C3G [2, 4].

Adipocytes are the main source of synthesis and secretion of a serine protease called adipsin or factor D (FD) [14–16]. This enzyme is considered the key limiting factor for the activation of the AP. FD cleaves factor B (FB) when it forms part of the C3 pro-convertase (C3bB), generating the active AP C3 convertase. Moreover, adipocytes also express other components of the AP, such as C3, FB and complement regulators like properdin (P), factor H (FH) and factor I (FI) [17–20]. Furthermore, local complement activation appears to be involved in the synthesis of triglycerides and adipocyte differentiation [17]. Although the connection between complement abnormalities and renal disease has been established, the exact mechanism of fat loss remains unclear. Mathieson and collaborators demonstrated that C3NeF could induce complement-mediated lysis of adipocytes in vitro [21]. However, the fact that only a small proportion of patients with C3NeF develop lipodystrophy remains unexplained.

We herein describe the immunological and clinical characteristics of a group of 13 patients diagnosed with BSS. Our results show that C3 hypocomplementemia and C3NeF autoantibodies are present in approximately 70% of the patients; besides, we found the presence of other autoantibodies against individual complement components. Immunological studies were extended with Human Leukocyte antigen (HLA) phenotyping and the screening of autoimmune markers.

## Results

### Demographic and clinical data

Demographic details and basic clinical information are listed in Table 1. In our cohort, patients were mainly female (ratio 5.5:1) and the mean age at the time of the study was 33 (range 8–76). Lipodystrophy onset occurred during childhood (mean 8 years) in most cases and only one patient, BSS6, developed the disease in adulthood (41 years old) after the implantation of orthodontic brackets. Most of the patients conserved the body mass index (BMI) into normal range, and some them registered slight decreases of the percentage of whole total fat mass. Regarding glucidic metabolism, one patient had elevated insulin and HbA1c levels and was diagnosed with diabetes. Mild low leptin levels (8.03 ng/ml; normal range: 15.3 ± 8.1 standard deviation) were present in one patient and triglycerides, total cholesterol and LDL-cholesterol levels were elevated in two patients. Three patients were diagnosed with fatty liver, linked to higher concentrations of AST and ALT transaminases. BSS1 suffered a hepatitis B virus (HBV) infection that lead to the deterioration of liver function with elevation of transaminases and development of hepatocellular carcinoma which shortly ended in death. Concerning cardiovascular diseases, three patients developed arterial hypertension. Finally, BSS1, BSS3 and BSS9 were diagnosed with DDD, IgA nephropathy and C3GN, respectively.

**Table 1:** Clinical and demographical data of patients with BSS

Variable	BSS1	BSS2	BSS3	BSS4	BSS5	BSS6	BSS7	BSS8	BSS9	BSS10	BSS11	BSS12	BSS13	Reference
Sex	F	F	F	F	F	F	F	F	M	M	F	F	F	
Age (years)	76	13	15	8	49	42	41	44	52	11	62	10	12	
Lipodystrophy onset (years)	N/A	2	5	4	9	41	7	3	6	7	8	7	7	
BMI (kg/m2)	24.3	19.3	18	16.3	19.8	22.3	33.8	22.7	23.5	21.3	26.1	16.5	22.7	18–25
% total fat^a^	N/A	N/A	17.7	22	20.3	27.8	41.8	23	28.7	33.2	29.1	31.2	42.6	25–31 (Women) 18–24 (Men)
Acanthosis	No	No	No	No	No	No	No	No	No	No	No	No	No	Yes/No
Glucose (mg/dL)	79	85	69	86	83	93	82	102	84	90	107	94	84	60–110
Insulin (μU/mL)	15.8	10.4	10.7	13.5	N/A	N/A	18.5	9.8	13	14.3	33.3	9	4.74	2.6–24.9
Diabetes	No	No	No	No	No	No	No	No	No	No	Yes	No	No	Yes/No
HbA1c (%)	N/A	4.8	N/A	5.3	5.2	N/A	5.4	N/A	N/A	5.2	6.4	5.1	5.2	4.0–5.5
Hypoleptinemia	No	No	Yes	No	No	No	No	No	No	No	No	No	No	Yes/No
Triglycerides (mg/dL)	106	79	110	89	78	66	105	210	445	61	265	79	53	25–115
Total colesterol (mg/dL)	129	145	122	163	238	183	158	120	245	112	175	167	159	110–230
HDL-cholesterol (mg/dL)	57	62	N/A	42	69	70	63	35	45	45	37	44	66	40–70
LDL-cholesterol (mg/dL)	120	109	N/A	112	153.4	100	74	111	163	64	110	126	101	50–129
Fatty liver^b^	No	No	No	No	No	No	Yes	No	No	Yes	Yes	No	No	Yes/No
ALT (UI/L)	33	28	13	24	31	20	60	21	21	15	24	22	28	5–31
AST (UI/L)	51	24	23	33	26	19	39	15	15	24	25	25	20	5–32
Hypertension	Yes	No	No	No	No	No	No	No	Yes	No	Yes	No	No	Yes/No
Renal disease	Yes (DDD)	No	Yes (IgAN)	No	No	No	No	No	Yes (C3GN)	No	No	No	No	Yes/No

Abbreviations: *F* female, *M* male, *BMI* Body Mass Index, *HbA1c* Glycohemoglobin, *ALT* Alanine aminotransferase, *AST* Aspartate aminotransferase; *DDD* Dense deposit disease, *IgAN* IgA nephropathy, *C3GN* C3 glomerulonephritis, *N/A* Not available

^a^ Percentage of total fat was measured using dual-energy x-ray absorptiometry

^b^ Liver steatosis was assessed by means of ultrasonography

### Complement system profile in patients with BSS

**Fig. 1:**
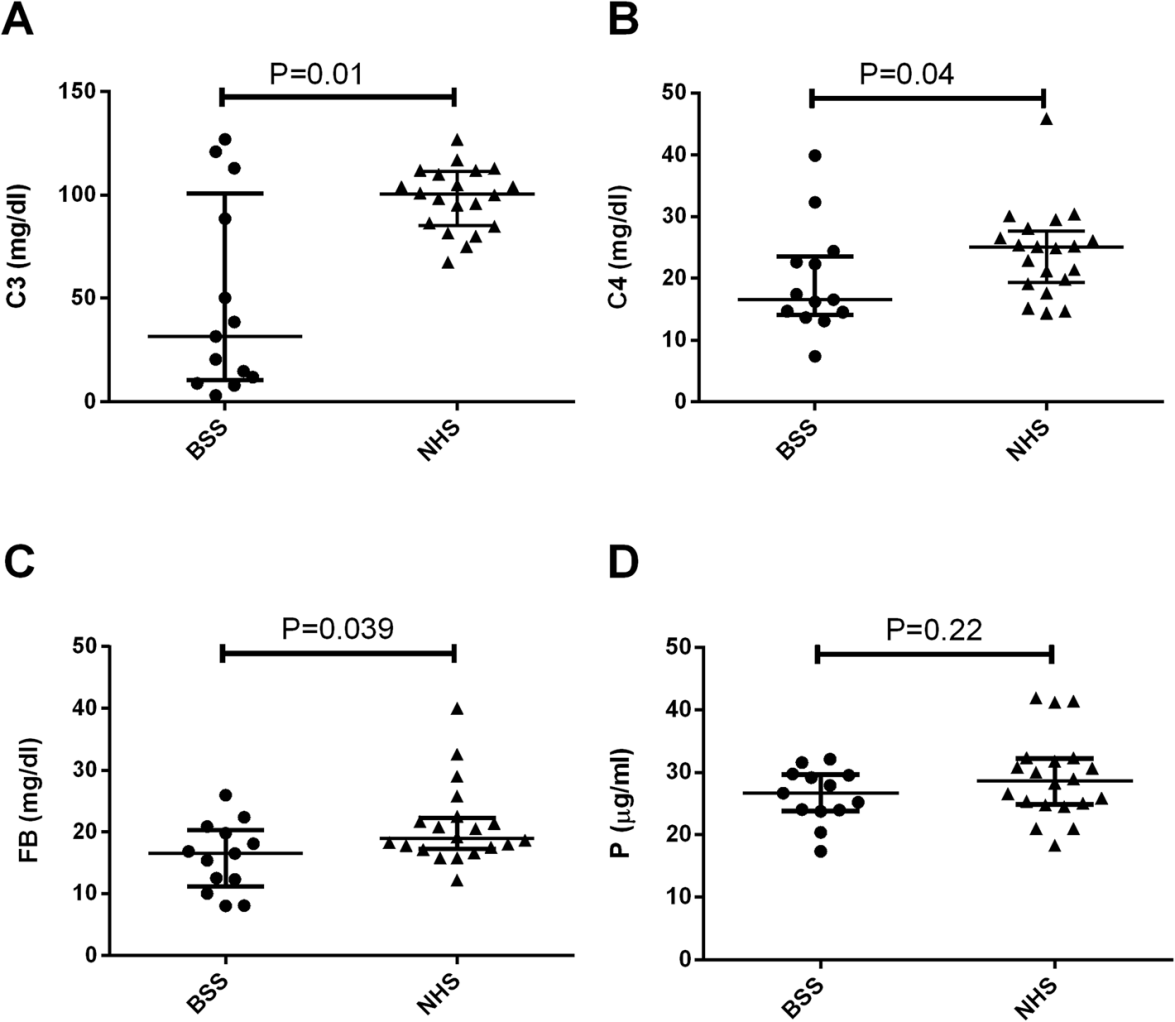
Complement profile in Barraquer-Simons syndrome (BSS) cohort. (**a**) C3 levels, (**b**) C4 levels (**c**) Factor B (FB) levels and (**d**) properdin (P) levels. Data are represented by median with interquartile ranges. The results of the BSS cohort (*n* = 13) are compared with a group of 20 healthy subjects (NHS). Data is statistically significant for *P* < 0.05

C3 levels were significantly reduced (*P* = 0.01) in the group of patients with BSS [median 31.7 mg/dl (IQR, 10.4–100.8)] in comparison with healthy donors (NHS) [100.5 mg/dl (85.2–111.5)] (Fig. 1a). The same applies for C4 [BSS, 16.5 mg/dl (14.1–23.5); NHS, 25 mg/dl (19.3–27.7); *P* = 0.04] and FB levels [BSS, 16.5 mg/dl (14.1–23.5); NHS, 25 mg/dl (19.3–27.7); *P* = 0.04] (Fig. 1b), that presented significant differences between both groups.

Conversely, similar P levels were found in controls and BSS patients [BSS, 26.7 mg/dl (23.9–29.7) versus NHS, 28.7 mg/dl (24.9–32.3), *P* = 0.22] (Fig. 1d).

### C3NeF is not the only autoantibody against AP components in patients with BSS

The frequencies of these autoantibodies are shown in Table 2. C3NeF was the most frequent autoantibody (69.2% of cases) observed in sera from patients with BSS, in accordance with previous observations [2, 4, 5]. Additional autoantibodies against the AP C3 convertase (C3bBbP) components were also detected. Autoantibodies against P were the most prevalent (38.5% of cases), followed by anti-C3 and anti-FB autoantibodies (30.8% for both). Moreover, the presence of one or more of these autoantibodies was always associated with C3 consumption. However, none of them showed significant reactivity against complement negative regulators FH and FI. Autoantibodies against complement proteins were not found in the 20 healthy subjects analyzed as control population. A schematic representation of these autoantibodies and their antigens is shown in Additional file 1: Figure S1.

**Table 2:** Frequencies of autoantibodies against complement proteins in patients affected by BSS

	C3NeF	anti-C3	anti-FB	anti-P	anti-FI	anti-FH	C3 (mg/dl)
BSS1	Positive	Positive	Negative	Negative	Negative	Negative	3.0
BSS2	Positive	Positive	Negative	Negative	Negative	Negative	11.9
BSS3	Positive	Positive	Positive	Positive	Negative	Negative	31.7
BSS4	Positive	Positive	Negative	Negative	Negative	Negative	7.9
BSS5	Positive	Negative	Negative	Negative	Negative	Negative	14.8
BSS6	Negative	Negative	Negative	Negative	Negative	Negative	88.5
BSS7	Positive	Negative	Positive	Positive	Negative	Negative	38.7
BSS8	Negative	Negative	Negative	Negative	Negative	Negative	121.0
BSS9	Positive	Negative	Positive	Positive	Negative	Negative	20.6
BSS10	Positive	Negative	Positive	Positive	Negative	Negative	50.2
BSS11	Negative	Negative	Negative	Negative	Negative	Negative	127.0
BSS12	Positive	Negative	Negative	Positive	Negative	Negative	8.9
BSS13	Negative	Negative	Negative	Negative	Negative	Negative	113.0
	9/13 (69.2%)	4/13 (30.8%)	4/13 (30.8%)	5/13 (38.5%)	0/13 (0%)	0/13 (0%)	Range: 75–135 mg/dl

Abbreviations: *C3NeF* C3 nephritic factor, *FB* factor B, *P* properdin, *FI* factor I, *FH* factor H

### Profile of HLA alleles in patients with BSS

To investigate the immunogenetic variables potentially associated with BSS we determined the HLA class I and class II phenotypes (Table 3). The very low sample size and the enormous polymorphism of the HLA system preclude a proper, statistically powered, study of association. However, examination of phenotypes reveals that: (i) no HLA allele was shared by all, or a vast majority of BSS patients; (ii) notwithstanding, 3/9 Spanish and 1/4 Italian patients (ca. 31%) carried allele DRB1*11:03, reported in Spaniards at an allelic frequency of ~ 1.3% (calculated carrier frequency ~ 2.6% [22]), the global frequency of all DRB1*11 alleles in BSS patients being 54%; and (iii), no patient carried B*07, B*14 and DRB1*15 alleles, all common in European Caucasoids. Future studies in larger patients’ cohorts should address whether these observations reflect a real predisposing effect.

**Table 3:** HLA profiles of patients affected by BSS

Patient							
BSS1	A*01:01,*30:02	B*08:01,*18:01	C*05:01,*07:01	DRB1*03:01	DQB1*02:01	DQA1*05:01	DPB1*01:01,*02:02
BSS2	A*02:01,*11:01	B*35:01,*57:03/B*35:04,*57:01	C*04:01,*07:01	DRB1*11:03,*14:54	DQB1*03:01,*05:03	DQA1*01:04,*05:05/09	DPB1*04:01
BSS3	A*03:01,*11:01	B*27:05,*35:01	C*01:02,*04:01	DRB1*01:01,*04:03	DQB1*03:02,*05:01	DQA1*01:01,*03:01	DPB1*04:01
BSS4	A*02:01,*24:02	B*40:06,*49:01	C*07:01,*15:02	DRB1*01:01,*11:03	DQB1*03:01,*05:01	DQA1*01:01,*05:05/09	DPB1*02:01,*04:01
BSS5	A*03:01,*29:02	B*18:01,*44:02	C*05:01	DRB1*03:01,*11:01	DQB1*02:01,*03:01	DQA1*05:01,*05:05/09	DPB1*04:01
BSS6	A*11:01,*24:02	B*53:01,*57:01	C*04:01,*06:02	DRB1*07:01,*13:02	DQB1*03:03,*06:04	DQA1*01:02,*02:01	DPB1*03:01,*04:02
BSS7	A*29:02,*68:01	B*44:02,*49:01	C*07:01,*07:04	DRB1*04:01,*07:01	DQB1*02:02,*03:01	DQA1*02:01,*03:01	DPB1*04:01
BSS8	A*02:01,*24:02	B*15:01,*41:02	C*03:03,*17:01/17:03	DRB1*11:03,*13:03	DQB1*03:01	DQA1*05:05/09	DPB1*04:01,*04:02
BSS9	A*32:01,*68:01	B*35:08,*51:01	C*04:01,*15:02	DRB1*04:03,*13:01	DQB1*03:02,*06:03	DQA1*01:03,*03:01	DPB1*02:01,*04:01
BSS10	A*02:01,*24:02	B*15:01,*18:01	C*03:03,*12:03	DRB1*04:01,*11:04	DQB1*03:01,*03:02	DQA1*03:01,*05:05/09	n.s.
BSS11	A*02:01,*24:02	B*35:01,*45:01	C*04:01,*07:02	DRB1*11:01,*11:03	DQB1*03:01	DQA1*05:05/09	n.s.
BSS12	A*02:01	B*27:05,*39:06	C*02:02,*07:02	DRB1*08:01,*13:01	DQB1*04:02,*06:03	DQA1*01:03,*04:01	n.s.
BSS13	A*02:01,*11:01	B*44:02,*55:01	C*03:03,*05:01	DRB1*10:01,*16:01	DQB1*05:01,*05:02	DQA1*01:01/04/05,*01:02	n.s.

The most likely HLA alleles at four-digit resolution, assigned as indicated in Materials and Methods, are displayed for each patient. Slashes represent ambiguities in the phenotype; *n.s.* not studied

### Autoimmunity and autoimmune diseases are prevalent in patients with BSS

The profile of autoantibodies and related diseases are summarized in Table 4**.** Clinical data from the 13 patients showed a high prevalence of autoimmune diseases (38.5%) including Hashimoto’s thyroiditis (*n* = 2)**,** vitiligo (*n* = 1), Sjögren’s syndrome (*n* = 1) and rheumatoid arthritis (*n* = 1). Moreover, 61.5% of patients were positive for one or more autoantibodies tested and ANA were detected in 30.8% at a 1/160 titer in all cases.

**Table 4:** Clinical and laboratory markers of autoimmunity in patients affected by BSS

	Autoimmune markers	Autoimmune disease
BSS1	Anti-dsDNA indeterminated, RF	Rheumatoid arthritis
BSS2	–	–
BSS3	–	–
BSS4	–	–
BSS5	TG-Ab	Hashimoto’s thyroiditis
BSS6	–	–
BSS7	TG-Ab, TPO-Ab	Hashimoto’s thyroiditis, vitiligo, Sjögren’s syndrome
BSS8	–	–
BSS9	ANA (1:160)	–
BSS10	DAT+	–
BSS11	ANA (1:160)	–
BSS12	DAT+, ENA (Anti Ro52Ab), ANA (1:160)	–
BSS13	APCA, ANA (1:160), GAD-Ab	–

Abbreviations: *Anti-dsDNA* anti-double stranded DNA, *RF* rheumatoid factor, *TG-Ab* thyroglobulin antibody, *TPO-Ab* thyroid peroxidase antibody, *ANA* anti-nuclear antibody, *DAT* Direct Antiglobulin Test, *APCA* anti-parietal cell antibody, *GAD-Ab* Glutamic Acid Decarboxylase autoantibodies; −, absent

Two patients (BSS5 and BSS7) who were diagnosed with Hashimoto’s thyroiditis were positive for anti- TG-Ab and one of them (BSS7) for TPO-Ab. Patients BSS10 and BSS12 were positive for DAT or Coombs test but they were not affected with autoimmune hemolytic anemia at the time the study was performed. Another patient with Hashimoto’s thyroiditis also presented with vitiligo and Sjögren’s syndrome (BSS7). One patient had active rheumatoid arthritis with elevated rheumatoid factor (BSS1). APCA and Glutamic Acid Decarboxylase GAD-Ab were found in one patient (BSS13) but no related diseases have been developed at present. No dermatomyositis-related autoantibodies were detected in any patient.

## Discussion

Abnormalities in the AP of the complement system are highly frequent in patients with BSS [2, 3, 5, 8]. Presumably as a consequence of AP dysregulation, C3 hypocomplementemia has been reported in 70–80% of patients with BSS [3]. Moreover, this feature is widely established as a critical marker for the differential diagnosis of this type of lipodystrophy [4].

In the present study and in line with previous reports, C3 hypocomplementemia was found in 69.3% of the patients all of whom were positive for C3NeF (Fig. 1a) [5, 8]. Furthermore, BSS patients in our series also had significantly low FB levels as compared to controls (Fig. 1c). FB is a complement protein subjected to proteolytic cleavage by FD during the activation of the AP. FB levels may indeed be reduced in patients with C3NeF but this is not a constant finding. This issue has been debated in several works but there are heterogeneous results concerning FB levels in the published series [23–25]. During AP activation, in the absence of C3NeF, C3 and FB consumption are usually correlated; however, C3NeF breaks this correlation. One molecule of FB is needed for the assembly of one AP C3 convertase. C3NeF stabilizes the C3 convertase so that thousands of C3 molecules are proteolyzed by this complex leading to C3 consumption with no need for further convertase formation and subsequent FB consumption. This stabilizing effect of C3NeF is heterogeneous among individuals, so it may be hypothesized that the decrease of FB levels is correlated with the stabilizing ability of the heterogeneous C3NeF pool in each patient. Strikingly, P levels remained normal in our cohort (Fig. 1d). One possible explanation is that P is not the target of proteolytic cleavages and its consumption during the activation of the AP is unlikely.

Activation of the classic pathway (CP), resulting in low C4, has been described in some patients with acquired generalized lipodystrophy (AGL), though not being considered an specific feature of this pathology [26, 27]. In our study, C4 levels were significantly lower in BSS patients compared to controls (Fig. 1b**).** This observation suggests that, in BSS, as previously described, the activation of both CP and AP may occur [8]. The activation of CP could be explained as a consequence of the formation of antigen-antibody complexes. Interestingly, in patients who tested positive for the presence of antibodies against C3 (BSS1 to BSS4, see Table 2), one of the most abundant plasmatic proteins, lower C4 levels were found, probably as a consequence of the higher titers of circulating immune-complexes. However, further studies are needed to confirm this association.

In this report, age- and sex-matched healthy subjects were not available as controls, which is a limitation of the study. Other authors have previously shown that complement protein levels modify during aging; however, C3, C4, FB or P (proteins quantified in this work) do not exhibit significant variation with aging [28] and linear regression studies performed on our data have confirmed that aging is not influencing the results of the study (Additional file 1: Figure S2). Moreover, previous reports have established the normal variation range of C3 and C4 complement levels with age [29], and data presented here for patients with BSS are out of normal ranges, again indicating that the observed variation is not due to aging.

Females were overrepresented in both our patient and control cohorts (85 and 60%, respectively). According to Gaya da Costa et al. [28], the levels of C3 and P are also significantly lower in women in comparison with men, which would result in significant bias in quantitative studies. To discard such effect, we compared the levels of C3, C4, FB, and P between sexes, and no significant differences for any complement component were found in our cohort of healthy subjects (Additional file 1: Figure S3).

Our results demonstrate an association between the presence of autoantibodies directed against proteins of the AP and altered complement levels in patients with BSS. All of those patients with BSS and severe C3 consumption were positive for at least one of the analyzed autoantibodies the more prevalent being C3NeF (Table 2). C3NeF is associated with a predisposition to develop kidney disease in the medium to long term, which may explain why some patients in our series have developed C3G (BSS1 and BSS9) and IgA nephropathy (BSS3) (Table 1). C3G and IgAN are two diseases with different pathophysiological bases that share marked dysregulation of the complement system. Although associations between IgAN and glomerular and circulating markers of complement activation are established, the mechanism of complement activation and contribution to glomerular inflammation and injury is not defined. All reports had described that C3G is the main cause of renal disease in patients with BSS and in these cases activation of the AP is the main mechanism involved in complement dysregulation. In the IgAN, lectin and AP could be involved in the activation of the complement system. This case with IgAN and BSS could be the result of an association of two entities in which complement consumption should be mostly related with the presence of an IgG with C3NeF activity. However, we cannot rule out this to be an accidental finding not related to the lipodystrophic phenotype.

Regarding the antibodies against additional complement proteins (C3, FB and P) it should be noted that they were detected only in patients who were also positive for C3NeF (Table 2). Accordingly, it is reasonable to speculate that whatever the mechanism leading to the break of immunological tolerance, if the C3bBb convertase (with or without P) bears the neoepitope targeted by C3NeF, additional autoantibodies directed against single proteins of this complex could be concurrently generated. Of note, autoantibodies against negative regulators of the AP (FH and FI) were not found in these patients (Table 2). Unlike C3NeF, whose importance in lipodystrophy has been largely studied and validated, autoantibodies against individual components of the C3 convertase have never been previously described in BSS. Anti-FB autoantibodies were first reported in a patient with DDD by Strobel and coleagues [30]. Their study revealed that they prevented the spontaneous dissociation of the C3 convertase of the AP and increased its normal half-life, thus causing systemic complement activation in the patient [30]. Additional patients with anti-FB and anti-C3b autoantibodies have since been reported in C3G and Ig-Associated Membranoproliferative Glomerulonephritis cohorts supporting their pathological role in the dysregulation of the AP [31, 32]. Therefore, further studies are needed in order to clarify if anti-FB and anti-C3b autoantibodies have a primary mechanistic role in the pathology of the lipodystrophy or if they rather secondarily arise as a consequence of the increase in circulating complement proteins produced due to unabated complement activation in the presence of C3NeF. A plausible possibility in line with the presence of C3NeF and low C3 levels in our BSS cohort is that these autoantibodies synergistically promote further C3 convertase stabilization and C3 consumption in serum, similarly to what has already been described by Vasilev and colleagues for anti-C3 and anti-C3b autoantibodies in patients with lupus nephritis [33, 34].

Although the etiology of BSS is generally unknown, several studies have described its association with signs of autoimmunity [5, 7, 35], a feature which is considered a supportive clinical criterion for the diagnosis to BSS [5]. Patients have been recruited in Italy and Spain, and autoantibody screening has been performed in both countries. To minimize the bias due to different methodology and interpretation, especially in the case of indirect immunofluorescence studies, autoantibody analysis was centralized in one laboratory from each country (La Paz University Hospital and University Hospital of Pisa). These determinations were performed in clinical routine laboratories with broad experience in autoimmunity and using validated methods. This study demonstrates a strong association of BSS with autoimmunity because 61.5% of the patients are positive for one or more autoantibodies, and 38.5% of them developed autoimmune-related disorders (Table 4). The patient BSS1 suffered from chronic HBV infection that could be associated with the presence of rheumatoid arthritis and markers of autoimmunity (RF and anti-dsDNA Ab). Several reports have illustrated the mechanisms involved in the loss of tolerance as a consequence of the immune response to HBV infection: such as molecular mimicry between HBV antigens and self-proteins, generation of immune complexes between HBV antigens and antibodies, promotion of apoptosis/tissue damage. All the above mentioned mechanisms culminate in the exposure of intracellular antigens to the immune system and may end up with the development of a variety of autoimmune diseases [36, 37].

Since the first description of anti-adipocyte autoantibodies in a patient with AGL by Hübler and colleagues [38], its existence in BSS has been discussed. The results of the present study provide additional supportive evidence for a different autoimmune etiological basis for BSS according to which either local or systemic complement activation induced by autoantibodies against the C3 convertase of the AP or its individual components may play a relevant role.

We hypothesized that genetic and/or environmental factors a role in the disease onset. Among the putative genetic factors, HLA region on chromosome 6 is a reasonable candidate, as demonstrated through strong associations with a large variety of autoimmune or inflammatory diseases [39] such as type 1 diabetes and celiac disease [40, 41], Juvenile Autoimmune thyroiditis or ankylosing spondylitis with HLA-B*27 association [42, 43]. Although no HLA allele was shared by the majority of our patients, interestingly, the allele DRB1*11:03 were overrepresented in our cohort (31% carrier frequency vs 1.3% allelic frequency in the general population) [22]) (Table 3). There are hundreds of polymorphisms of HLA-DRB1 which have been associated with different autoimmune disorders as well as with immune response to infection and vaccines The association between HLA-DRB1*11 and autoimmune, infectious and cancer diseases has been previously reported for: systemic sclerosis, Henoch-Schönlein purpura, systemic juvenile idiopathic arthritis, *Helicobacter pylori*-positive idiopathic thrombocytopenic purpura, hairy cell leukaemia, cervical cancer, among others [44–49]. There are at least two reports dealing with an apparent association of the allele DRB1*11:03 with forms of juvenile idiopathic arthritis [50, 51]. Future studies in larger patients’ cohorts should address whether this allele is a real biomarker of BSS or not.

## Conclusions

We here confirm that C3, and to a lower extent, C4 hypocomplementemia are common features of BSS, and that this pathology is frequently associated with autoimmunity. Moreover, besides C3NeF, other autoantibodies directed against components of the C3 convertase of the AP (anti-C3, anti-FB and anti-P) are present in a significant proportion of patients from our cohort. Finally, an association with the HLA allele DRB1*11:03 was observed, suggesting a potential role of this variant as a marker of the disease.

## Methods

### Patients

Thirteen patients were diagnosed with BSS on the basis of fat loss during childhood or adulthood affecting upper areas of the body, and having ruled out other causes of fat loss. The diagnosis was made based on standardized criteria [1]. The presence of other autoimmune diseases can be of support of the diagnosis. Laboratory findings as low serum C3 and the presence of C3NeF were used during the diagnosis. Familial Partial Lipodystrophy (FPL) was also excluded based on the natural course of the disease, clinical features, age at onset, and absence of pathogenic variants in FPL-related genes (*LMNA, PPARG, PLIN1, CIDEC, LIPE, ADRA2A, AKT2*). No consanguinity was reported in any case.

### Biological samples

We collected serum samples from 13 patients with BSS (9 from Spain, and 4 from Italy) and 20 healthy subjects (NHS) (60% female, aged 25–61 years). Controls and patients are not matched for age. Serum and EDTA plasma samples were obtained under standard conditions upon informed consent from the donors; blood was collected in plain tubes, allowed to clot at room temperature, and centrifuged for 10 min at 4 °C. Serum and plasma were then aliquoted and stored frozen at − 80 °C until their use.

### Measurement of the levels of complement system proteins

Serum C3 and C4 levels were measured by nephelometry (Siemens Healthcare, Erlangen, Germany). FB and P levels were measured using in-house ELISA assays, previously described by our group [23].

### C3NeF detection by enzyme-linked immunosorbent assay (ELISA)

C3NeF detection in serum samples was performed as described previously by Paixão- Cavalcante and collaborators [24] with several modifications. Briefly, residual Bb was detected using a monoclonal anti-Bb antibody (A227, Quidel) (1:500; 1 h, 37 °C), followed by peroxidase-conjugated goat anti-mouse IgG (Jackson Immunoresearch, West Grove, PA, USA) (1:5000; 1 h). Color was developed using *o*-phenylenediamine dihydrochloride (Sigma-Aldrich, Madrid, Spain) and absorbance was measured at 492 nm. Samples were considered positive when optical density was higher than 0.3 units of absorbance.

### Detection of autoantibodies against alternative pathway components (C3, FB and P) and regulators (FI and FH) by ELISA

ELISA plates were coated with 100 ng/well of purified C3, FB, FI, FH, or P. Plates were blocked with PBS-BSA 3% in case of FB and P, and with PBS-BSA 0.1% in FI and C3. Serum samples were diluted in PBS-BSA 0.1%, and binding of autoantibodies was detected with polyclonal anti-human IgG-HRP conjugated antibody (Jackson Immunoresearch) in ABTS substrate, as described by Nozal et al. [52]. Factor H autoantibodies were searched following methods previously described by Abarrategui-Garrido et al. [53]

### HLA genotyping

HLA typing has been performed by two different laboratories. The methodology developed by each laboratory is described below:

- Spain: HLA-A, −B, −C, −DRB1, −DQB1 and -DQA1 were studied by polymerase-chain reaction with sequence-specific oligonucleotide probes (PCR-SSOP) using commercial reagents (details available upon request). Those reagents distinguish all two-digit allelic groups and also discriminate most four-digit alleles commonly seen in the Spanish population [22, 40, 54–56]. In addition, we determined DPB1 phenotypes and confirmed DRB1*11:03 by sequence-based typing using local reagents. All HLA typing studies were performed following the quality standards issued by the European Federation for Immunogenetics.

- Italy: A single PCR reaction was used for each HLA locus. Luminex-based technology was applied to discriminate among the different human HLA alleles, by using sequence-specific oligonucleotide probes bound to color-coded microbeads in order to identify HLA alleles encoded by the DNA sample [57]. A flow analyzer identifies the fluorescent intensity SAPE on each microsphere. A software was used to assign positive or negative reactions based on the strength of the fluorescent signal.

### Screening of autoantibodies

The screening of autoantibodies was performed by two different laboratories. The methodology developed by each laboratory is described below:

- Spain: Anti-cellular autoantibodies were tested by indirect immunofluorescence (IIF) on Hep-2 cells (Euroimmun, Luebeck, Germany), and ENA analysis was performed, either of IIF was positive or not, by Bioplex ANA Screen kit (Bio-Rad, Hercules, CA), which includes dsDNA, chromatin, centromere B, Scl70, RNP-68 kDa, RNP-A, Ro/SSA 52 kDa, Ro/SSA 60 kDA, La/SSB, Sm, Sm/RNP, Jo-1, and P ribosomal proteins as antigens. Rheumatoid factor (RF) was determined by nephelometry (Siemens Healthcare, Erlangen, Germany).

Anti-thyroid peroxidase (TPO-Ab) and anti-thyroglobulin autoantibodies (TG-Ab) were quantified by fluorescence enzyme immunoassay (EliA, Phadia-Thermo Fisher, Freiburg, Germany). Anti-islet cells antibodies and anti-suprarrenal cortex autoantibodies detection was performed by IIF on monkey pancreas and monkey suprarenal gland tissues (Immco Diagnostics, Buffalo, NY and Biosystems, Barcelona, Spain, respectively). Anti-parietal cells autoantibodies (APCA), anti-mitochondrial and anti-smooth muscle autoantibodies were tested by IIF on rat liver, kidney and stomach substrates (Euroimmun, Luebeck, Germany).

- Italy: TPO-Ab and TG-Ab (AIA, Tosoh Bioscience Griesheim, Germany) and Anti-transglutaminase autoantibodies (Thermo Fisher Scientific, Waltham, USA) were detected by fluorescence enzyme immunoassay. Glutamic Acid Decarboxylase Autoantibodies (GAD-Ab) were tested by radioimmunoassay (Ria Medipan, Berlin Germany). APCA (Elisa Orgentc Diagnostika, Mainz, Germany), and 21-Hydroxylase (21-OH) Autoantibodies (Elisa RSR Cardiff, U.K) were quantified using commercial ELISA kits. ANA and dsDNA were tested by IIF (Euroimmun, Luebeck, Germany). Finally, Direct Antiglobulin Test (DAT) was analyzed by Column Agglutination (Ortho Clinical Diagnostics Pencoed, UK).

### Statistical analysis

Statistical calculations were performed with Prism version 6.01 (GraphPad Software, La Jolla, CA, USA). Man-Whitney was used for comparisons among the groups. A *P*-value of < 0.05 was considered statistically significant in all analyses.

## Supplementary information

**Additional file 1: Figure S1.** The alternative pathway of the complement system and autoantibodies against its proteins. In the alternative pathway (AP), continuous, low-level activation of C3 by spontaneous hydrolysis of the internal C3 thioester, or C3 cleavage by plasma proteases, generates C3(H_2_O) or C3b. Activation by the AP leads to the generation of C3 convertase complexes (C3bBb) that cleave C3 into C3a and C3b. Additionally, the AP C3 convertase can bind properdin (P), a positive regulator that stabilizes the enzyme, extending its half-life more than 10-fold. The activation of the AP is controlled by two different soluble regulatory proteins, as factor H (FH) and factor I (FI). C3 nephritic factor (C3NeF) is one of the most known autoantibodies which recognized a neo-epitope in the C3 convertase. Other autoantibodies against complement components (C3, FB or P) and regulators (FH or FI), some of them with functional activities, have been shown in the figure. **Figure S2.** Lack of correlation between complement levels and age. In 20 healthy subjects no correlations were found among age and the levels of **(A)** C3, **(B)** C4 **(C)** factor B (FB) and **(D)** properdin (P). These data were obtained using the Spearman Rank correlation coefficient. *P* < 0.05 were considered statistically significant. **Figure S3.** Gender does not significantly modify complement levels in our cohort of healthy subjects. In 20 healthy subjects complement levels of **(A)** C3, **(B)** C4, **(C)** factor B (FB), and **(D)** properdin (P) were measured. The mean of concentration in males and females was analyzed by the Mann Whitney test. *P* < 0.05 was considered statistically significant.

## Data Availability

The data that support the findings of this study are available from the corresponding author upon reasonable request.

## Abbreviations

AGL: Acquired generalized lipodystrophy
AP: Alternative pathway
APCA: Anti-parietal cells autoantibodies
BSS: Barraquer-Simons syndrome
C3G: C3 glomerulopathy
C3GN: C3 glomerulonephritis
C3NeF: C3 nephritic factor
DAT: Direct Antiglobulin test
DDD: Dense deposit disease
FB: Factor B
FD: Factor D
FH: Factor H
FI: Factor I
FPL: Familial Partial Lipodystrophy
GAD-Ab: Glutamic Acid Decarboxylase Autoantibodies
HBV: Hepatitis B Virus
HLA: Human Leukocyte antigen
P: Properdin
RF: Rheumatoid factor
TG-Ab: Anti-thyroglobulin autoantibodies
TPO-Ab: Anti-thyroid peroxidase
