# Correction to: Immunological features of patients affected by Barraquer-Simons syndrome

**DOI:** 10.1186/s13023-020-1350-8

**Published:** 2020-03-30

**Authors:** Fernando Corvillo, Giovanni Ceccarini, Pilar Nozal, Silvia Magno, Caterina Pelosini, Sofía Garrido, Alberto López-Lera, Manuela Moraru, Carlos Vilches, Silvia Fornaciari, Sabrina Gabbriellini, Ferruccio Santini, David Araújo-Vilar, Margarita López-Trascasa

**Affiliations:** 1 Complement Research Group, Hospital La Paz Institute for Health Research (IdiPAZ), La Paz University Hospital, Paseo de la Castellana, 261, 28046 Madrid, Spain; 2 Center for Biomedical Network Research on Rare Diseases (CIBERER U754), Madrid, Spain; 3 Obesity and Lipodystrophy Centre at the Endocrinology Unit, University Hospital of Pisa, Pisa, Italy; 4 Unit of Immunology, La Paz University Hospital, Madrid, Spain; 5 Immunogenetics and Histocompatibility, Instituto de Investigación Sanitaria Puerta de Hierro, Madrid, Spain; 6 Immunogenetics laboratory, University Hospital of Pisa, Pisa, Italy; 7 Thyroid and Metabolic Diseases Unit (U.E.T.eM.), Centro Singular Investigación en Medicina Molecular e Enfermidades Crónicas (CIMUS-School of Medicine, Universidad de Santiago de Compostela, Santiago Compostela, Spain; 8 Universidad Autónoma de Madrid, Madrid, Spain

**Correction to: Orphanet J Rare Dis**

**https://doi.org/10.1186/s13023-019-1292-1**

Following the publication of the original article [1], the authors requested to amend the Abstract and Discussion section as follows:

**Abstract**

“The HLA allele DRB1*11 was present in 54% of BSS patients, and the majority of them (31%) were positive for *11:03 (vs 1.3% in the general population)”

has been amended to read:

“The HLA allele DRB1*11 was present in 54% of BSS patients, and the majority of them (31%) were positive for *11:03 (vs 1.3% **allelic frequency** in the general population)”.

**Discussion**

“Although no HLA allele was shared by the majority of our patients, interestingly, the allele DRB1*11:03 were overrepresented in our cohort (31% vs 1.3% in the general population)”

has been amended to read:

“Although no HLA allele was shared by the majority of our patients, interestingly, the allele DRB1*11:03 were overrepresented in our cohort (31% **carrier frequency** vs 1.3% **allelic frequency** in the general population)”.

The correction was deemed necessary to prevent possible misinterpretation of data deriving from the bibliography used. The original article has also been updated as above.
