# Supplementary material for: Immunological features of patients affected by Barraquer-Simons syndrome
Source: Orphanet J Rare Dis. 2020 Jan 10;15:9. doi: 10.1186/s13023-019-1292-1 (PMC6954565; doi:10.1186/s13023-019-1292-1)
Supplement: Supplementary file 1 — Additional file 1: Figure S1. The alternative pathway of the complement system and autoantibodies against its proteins. In the alternative pathway (AP), continuous, low-level activation of C3 by spontaneous hydrolysis of the internal C3 thioester, or C3 cleavage by plasma proteases, generates C3(H2O) or C3b. Activation by the AP leads to the generation of C3 convertase complexes (C3bBb) that cleave C3 into C3a and C3b. Additionally, the AP C3 convertase can bind properdin (P), a positive regulator that stabilizes the enzyme, extending its half-life more than 10-fold. The activation of the AP is controlled by two different soluble regulatory proteins, as factor H (FH) and factor I (FI). C3 nephritic factor (C3NeF) is one of the most known autoantibodies which recognized a neo-epitope in the C3 convertase. Other autoantibodies against complement components (C3, FB or P) and regulators (FH or FI), some of them with functional activities, have been shown in the figure. Figure S2. Lack of correlation between complement levels and age. In 20 healthy subjects no correlations were found among age and the levels of (A) C3, (B) C4 (C) factor B (FB) and (D) properdin (P). These data were obtained using the Spearman Rank correlation coefficient. P < 0.05 were considered statistically significant. Figure S3. Gender does not significantly modify complement levels in our cohort of healthy subjects. In 20 healthy subjects complement levels of (A) C3, (B) C4, (C) factor B (FB), and (D) properdin (P) were measured. The mean of concentration in males and females was analyzed by the Mann Whitney test. P < 0.05 was considered statistically significant. [file 13023_2019_1292_MOESM1_ESM.docx]

**
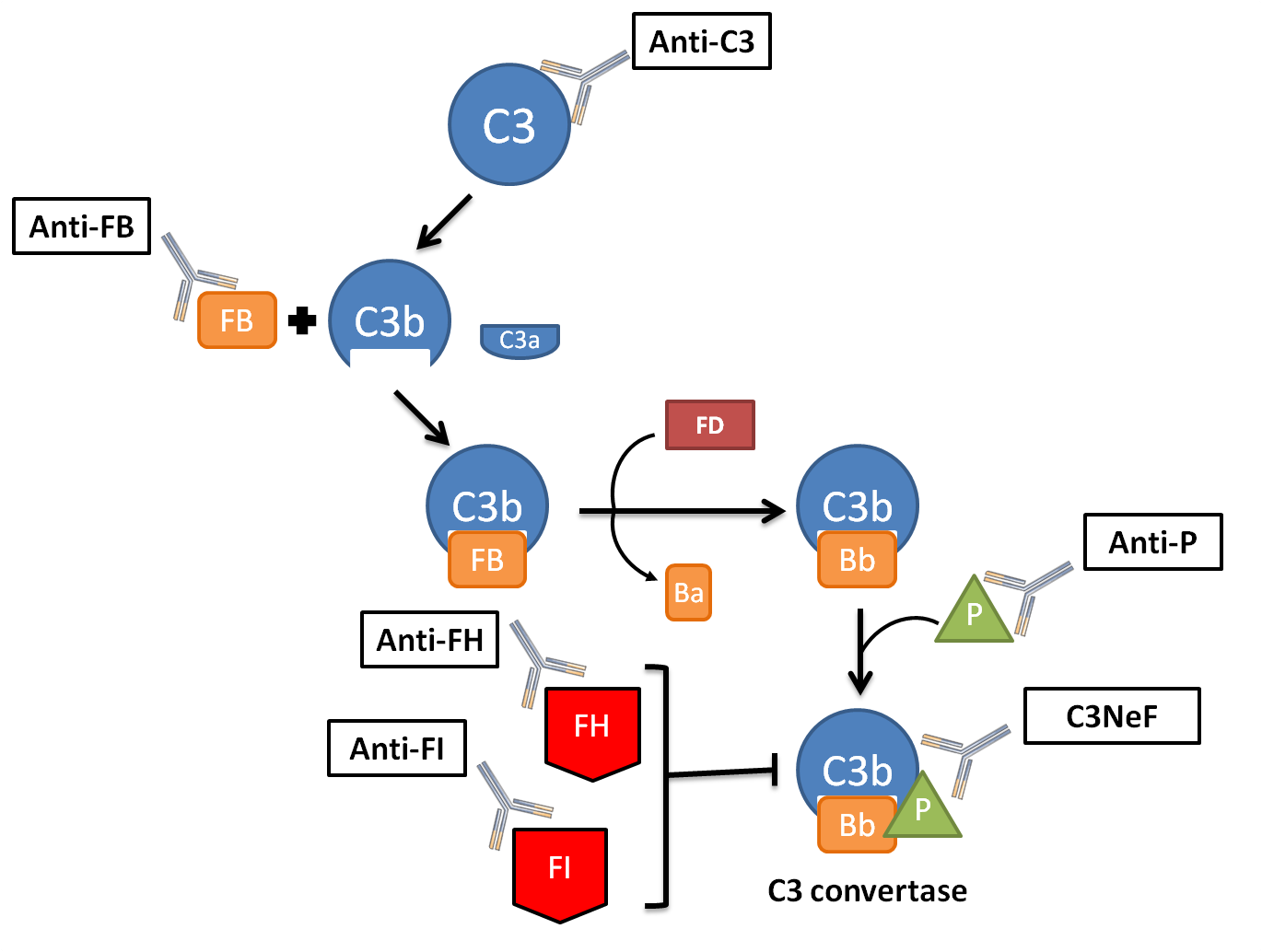
**

**Figure S1. The alternative pathway of the complement system and autoantibodies against its proteins.** In the alternative pathway (AP), continuous, low-level activation of C3 by spontaneous hydrolysis of the internal C3 thioester, or C3 cleavage by plasma proteases, generates C3(H_2_O) or C3b. Activation by the AP leads to the generation of C3 convertase complexes (C3bBb) that cleave C3 into C3a and C3b. Additionally, the AP C3 convertase can bind properdin (P), a positive regulator that stabilizes the enzyme, extending its half-life more than 10-fold. The activation of the AP is controlled by two different soluble regulatory proteins, as factor H (FH) and factor I (FI). C3 nephritic factor (C3NeF) is one of the most known autoantibodies which recognized a neo-epitope in the C3 convertase. Other autoantibodies against complement components (C3, FB or P) and regulators (FH or FI), some of them with functional activities, have been shown in the figure.





**Figure S2. Lack of correlation between complement levels and age.** In 20 healthy subjects no correlations were found among age and the levels of **(A)** C3, **(B)** C4 **(C)** factor B (FB) and **(D)** properdin (P). These data were obtained using the Spearman Rank correlation coefficient. *P* < 0.05 were considered statistically significant.





**Figure S3. Gender does not significantly modify complement levels in our cohort of healthy subjects.** In 20 healthy subjects complement levels of **(A)** C3, **(B)** C4, **(C)** factor B (FB), and **(D)** properdin (P) were measured. The mean of concentration in males and females was analyzed by the Mann Whitney test. *P* < 0.05 was considered statistically significant.
